# Unveiling the supramolecular assembly of a novel antimony(iii)-based co-crystal: integrating multi-spectroscopic analyses, DFT calculations, and advanced biological evaluations

**DOI:** 10.1039/d6ra01859a

**Published:** 2026-05-13

**Authors:** Nour Elleuch, Jerome Lhoste, Frédéric Amiard, Sergiu Shova, Mohamed Boujelbene, Sameh Sellami

**Affiliations:** a Laboratory of Physico-Chemistry of Solid State, LR11ES51, Sfax Faculty of Sciences, University of Sfax Sfax 3000 Tunisia m_boujelbene2010@yahoo.fr; b MMM-UMR 6283 CNRS, Lunam, Faculty of Sciences and Techniques, University of Maine Avenue Olivier Messiaen 72085 Le Mans Cedex 9 France; c “Petru Poni” Institute of Macromolecular Chemistry Alea Grigore Ghica voda 41-A 700487 Iasi Romania; d Biopesticides Laboratory, Centre of Biotechnology of Sfax, Sfax University P.O. Box ‘1177’ 3018 Sfax Tunisia

## Abstract

An antimony(iii) co-crystal [SbCl_3_(C_4_H_3_N_2_ClO)]_2_·(C_4_H_3_N_2_ClO)_2_ (abbreviated as PdzSb) was successfully prepared under hydrothermal reaction conditions at 140 °C and comprehensively analyzed through a variety of physicochemical techniques. The asymmetric unit comprises four distinct neutral species: two free ligands of 6-chloro-3(2*H*)-pyridazinone (6,3Pdz) and two discrete antimony-based complexes, each coordinated by three chloride ions and one aromatic ligand (C_4_H_3_N_2_OCl) (6,3Pdz–O) through the oxygen donor atom. Notably, the Sb(iii) center adopts a seesaw geometry, which is relatively uncommon and highlights the influence of the stereochemically active lone pair on the coordination environment. The main novelty of this work lies in establishing a clear correlation between the stereochemically active lone pair of Sb(iii), the resulting structural organization, and the associated physicochemical properties, rather than focusing on the optimization of a single specific application. Single-crystal X-ray analysis reveals that an extensive network of diverse non-covalent interactions plays a key role in stabilizing the three-dimensional supramolecular architecture, thereby governing the cohesion and properties of the co-crystal. Comprehensive spectroscopic and thermal analyses validated both the structural integrity and the chemical composition of the material. Hirshfeld surface and reduced density gradient (RDG) analyses emphasize the importance of non-covalent interactions, including hydrogen and halogen bonding, which are quantitatively evaluated using two-dimensional fingerprint plots. In addition, molecular electrostatic potential (MEP), electron localization function (ELF), and localized orbital locator (LOL) confirm the relevance of this interaction in the self-assembly process. To elucidate this unexpected observation and to further understand the intricate balance of the supramolecular interactions governing the crystal packing, a high-level density functional theory (DFT) study was conducted. Furthermore, optical studies indicate an indirect band gap of 3.42 eV, suggesting semiconducting behavior. Beyond its electronic functionality, the title compound exhibits significant antibacterial activity against clinically relevant human pathogens, with efficiencies comparable to those of standard antibiotics, and demonstrates promising larval biocontrol activity against lepidopteran species. Therefore, these findings clearly establish structure–property–activity relationships and highlight the multifunctional nature of the Sb(iii) co-crystal that combines optical and biological functionalities in a single material.

## Abstract 2

An antimony(iii) co-crystal [SbCl_3_(C_4_H_3_N_2_ClO)]_2_·(C_4_H_3_N_2_ClO)_2_ (abbreviated as PdzSb) was successfully prepared under hydrothermal reaction conditions at 140 °C and comprehensively analyzed through a variety of physicochemical techniques.

## Graphical abstract

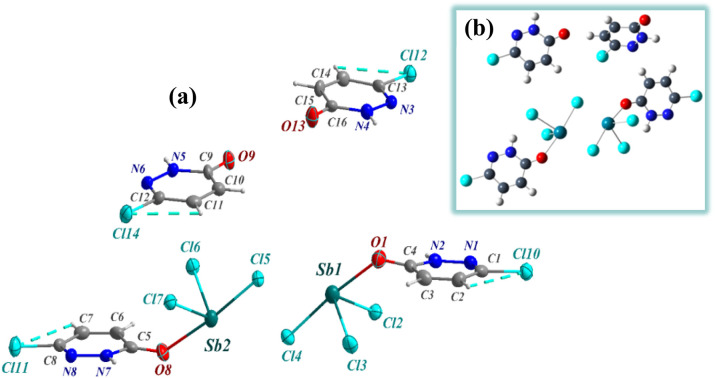

## 1. Introduction

Supramolecular co-crystal design, achieved through the non-covalent organization of simple molecular units,^1,2^ has emerged as a powerful and versatile approach for creating functional materials. In fact, the co-crystallisation process allows the stabilization of neutral molecules through a variety of noncovalent forces, including hydrogen bonding, π–π stacking, C–H⋯π interactions, and van der Waals forces,^3–5^ leading to the formation of innovative and multifunctional structures *via* the synergistic action of the constituent components. More recently, weak interactions, such as halogen, chalcogen, and pnictogen bonds, have become a highly debated topic^1,6–9^ as they play a central role in directing supramolecular assembly and modulating crystal packing. This latter role has significantly impacted the ability of chemists to design and construct structures of diverse sizes and shapes. Meanwhile, co-crystals represent a groundbreaking material, exhibiting novel physicochemical behaviors, including solubility, melting point, crystallinity, hygroscopicity, and chemical stability, beyond the reach of single materials.^10,11^ Although numerous organic co-crystals have been widely reported, multicomponent systems incorporating metal complexes remain comparatively less explored,^1,6,12,13^ despite their promising potential in medicinal and materials chemistry. In particular, metal-containing co-crystals may offer certain advantages over purely organic compounds across various fields of tremendous biological application, especially the pharmaceutical industry.^14^ Recent advances have revealed that antimony compounds exhibit a broad spectrum of coordination chemistry, as well as biological and cytotoxic activities,^15,16^ reflecting their importance in medicinal chemistry as antimicrobial, antitumor, antiviral (against the hepatitis C virus) and anti-cancer agents,^17–19^ although the detailed mechanisms of action remain insufficiently understood.^20^ Antimony, a member of group 15 (pnictogen) of the periodic table,^21^, is classified alongside other toxic heavy metals, and a well-known limitation of ‘heavy-metal therapy’ is the inherent toxicity of the metal. Nevertheless, the relevance of metal-based compounds in medicine is indisputable, as evidenced by the long-standing clinical use of antimony salts and ores to treat fevers and skin irritations since the ancient Egyptian era,^22^ as well as by their promising therapeutic potential against leishmaniasis.^19^ On the other hand, Sb(iii) can accept the lone pair of a donor ligand, acting as a Lewis acid toward a wide range of neutral N-, P-, O-, and S-donor ligands, providing stable complexes with different coordination numbers and structural diversity^23,24^ that have emerged as promising candidates for potential pharmaceutical applications.^18^ In addition to their established role in antimonial chemotherapy, antimony compounds have been investigated for their luminescent and semiconducting properties, highlighting their application potential in photovoltaic and optoelectronic devices.^25^ The biological and physicochemical properties of Sb(iii) complexes strongly depend on several factors, including their composition, solubility, reactivity, and oxidation state, as well as the stability and lability of the coordinated ligands.^17^ In this context, pyridazine derivatives constitute a particularly attractive class of ligands, owing to their established pharmacological versatility^26,27^ and their ability to act as neutral or anionic donors in metal coordination. Moreover, recent studies have highlighted that the non-fused pyridazine ring has emerged as a valuable heterocyclic motif in drug design, providing a versatile and ‘privileged’ scaffold. Ongoing research is aimed at exploring pyridazine-based small molecules that exhibit numerous significant biological activities, including anti-inflammatory,^28^ anti-diabetic,^29^ anti-obesity,^30^ neuroprotective,^31^ anti-Alzheimer's,^32^ anti-tubercular,^33^ anti-HIV,^34^ and anti-cancer^27^ activities. Notably, 3,6-dichloropyridazine is among the most reactive ligands owing to its strong tendency to form complexes with main group elements, particularly in unconventional co-crystalline materials. In turn, the presence of two chlorine atoms confers a strong reactivity toward nucleophilic aromatic substitution, with chlorides functioning as effective leaving groups.^35^ Instead of being eliminated as inert species, the displaced ligands possess biochemical relevance and may themselves serve as bioactive or medicinal agents. Additionally, 3,6-dichloropyridazine derivatives are of great interest as they can act as neutral or charged ligands, exhibiting tautomerism in keto/thione or enol/thiol form with coordination through oxygen, as reported for such complexes in the literature.^16^ To the best of our knowledge, only a single study has previously described a Bi(iii)-based co-crystal derived from 3,6-dichloropyridazine that displays antibacterial activity.^1^ Given the close chemical relationship between bismuth and antimony and their tendency to form extended supramolecular assemblies, further exploration of related Sb(iii) systems is both timely and scientifically relevant. In light of these considerations, the present work reports the hydrothermal synthesis, crystal structure and comprehensive physicochemical characterization of a new Sb(iii)-based co-crystal incorporating a pyridazinone ligand. Particular emphasis is placed on elucidating the supramolecular interactions governing crystal packing and on correlating these structural features with the optical and biological properties of the material, including antibacterial performance and larval biocontrol activity.

## 2. Experimental

### 2.1 Chemical preparation

**Scheme 1:**
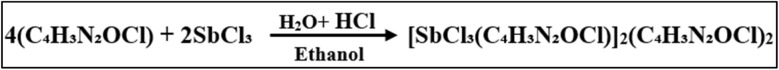
Reaction scheme illustrating the growth of the PdzSb co-crystal.

**Scheme 2:**
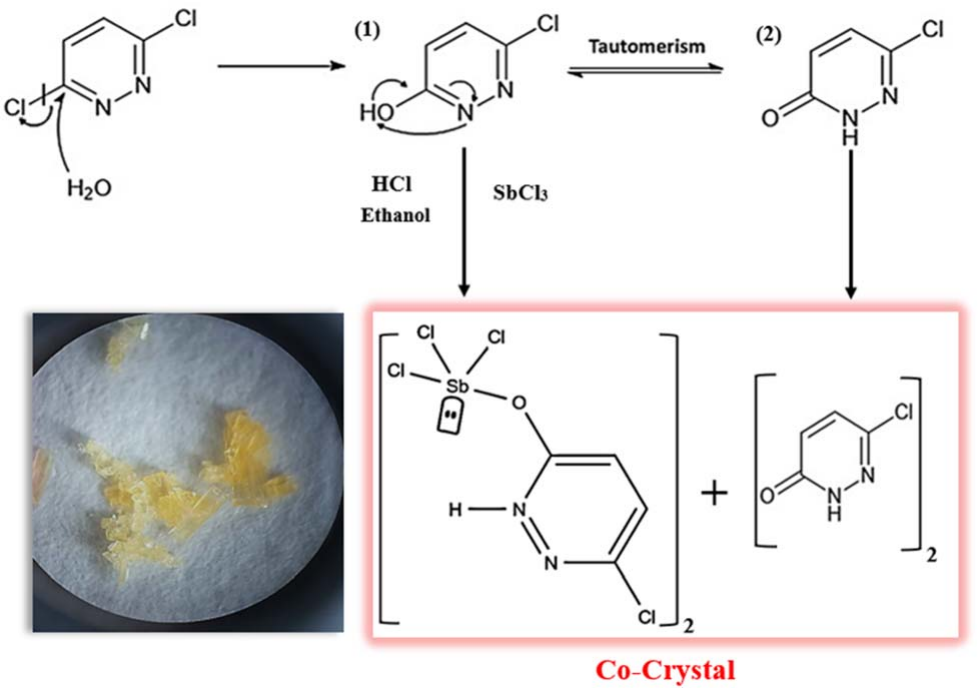
Reaction mechanism of the PdzSb co-crystal.

The antimony(iii)-based complex [SbCl_3_(C_4_H_3_N_2_OCl)]_2_ co-crystallized with (6,3Pdz–O)_2_ under hydrothermal conditions, leading to the formation of the PdzSb co-crystal. As reported in our previous work,^1^ 3,6-dichloropyridazine (purchased from Sigma-Aldrich) undergoes the same nucleophilic aromatic substitution (SN1) with water, which behaves as a nucleophile donor, yielding 3-chloro-6-hydroxypyridazine. In the present work, the resulting organic intermediate was combined in a 1 : 1 molar ratio with antimony trichloride (SbCl_3_), which was used in the earlier synthesis, under similar hydrothermal conditions (high pressure and temperature) for four days to afford the new antimony-based co-crystal. The chemical reaction scheme is expressed below (Scheme 1):

Following the mechanism described earlier,^1^ HCl promotes hydrolysis and pyridazine N-protonation, stabilizing the Sb coordination in 6,3Pdz–OSb. In turn, the neutral co-crystallizing species, 6-chloro-3(2*H*)-pyridazinone, is generated *via* tautomerization in protic polar solvents, involving an intramolecular enol–keto shift (Scheme 2 shown below).

### 2.2 X-ray data collection

The crystallographic data on PdzSb were collected at a temperature of 100 K using a Bruker SMART APEX-II CCD diffractometer with Mo Kα radiation (*λ* = 0.71073 Å). The structure was solved using SHELXT^36^ and refined by *F*^2^-based full-matrix least-squares refinement using SHELXL^37^ through the Olex2 software^38^ and the SHELX suite in the WinGX package.^39^ After several refinement cycles, the final refinement achieved *R*_1_ = 0.0281 and w*R*_2_ = 0.08, highlighting excellent structural retention. Anisotropic displacement parameters were applied to all non-hydrogen atoms, while hydrogen atoms were refined using a riding model. Specifically, all hydrogen positions were constrained with the HFIX instruction, assigning a value of 43 for both the NH and CH groups of the pyridazine ring. The structure exhibits inversion twinning, which was identified and appropriately taken into account during the refinement process. Molecular graphics, crystal-structure projections, and packing diagrams were generated using the DIAMOND software.^40^ The data collection and crystallographic parameters are given in Table 1. The selected interatomic distances and bond angles are presented in Table 2. Additional details of the crystallographic structure analysis are provided in the Crystallographic Information File (CIF), which has been deposited with the Cambridge Crystallographic Data Centre (CCDC) under deposition number 2449627.

**Table 1:** Crystallographic data and structure refinement details of PdzSb

Crystallographic data	
Chemical formula	[SbCl_3_(C_4_H_3_N_2_ClO)]_2_·(C_4_H_3_N_2_ClO)_2_
Formula weight (g mol^−1^)	978.34
Density calc. (mg m^−3^)	2.096
Color/shape	Clear light-yellow/plate
Crystal system	Orthorhombic
Space group	*Fdd*2
*a* (Å)	17.5960 (4)
*b* (Å)	28.4487 (7)
*c* (Å)	24.7684 (6)
*Z*	16
Volume (Å^3^)	12 398.6 (5)
Radiation du Mo	Mo Kα
Diffractometer	XtaLAB synergy, Dualflex, HyPix
Crystal size (mm^3^)	0.08 × 0.04 × 0.03 mm^3^
Temperature (K)	100
Wavelength (Å)	0.71073
Absorption coefficient *µ* (mm^−1^)	9.792
Index ranges	−20 ≤ *h* ≤ 17
	−32 ≤ *k* ≤ 33
	−27 ≤ *l* ≤ 29
Reflections collected	13 625
Independent reflections	5165
*θ* range for data collection (°)	2.6 < *θ* < 25.0
Reflections with *I* > 2*σ*(*I*)	4856
*F*(000)	7488
Number of refined parameters	362
Residual Fourier density (e Å^−3^)	−0.57 < Δ*ρ* < 1.44
w*R* (*F*^2^)	0.077
*R*[*F*^2^ > 2*σ*(*F*^2^)]	0.029
*R*_int_	0.025
S = GooF	1.04
CCDC	2449627

**Table 2:** Geometry of the hydrogen bonds of PdzSb

D–H⋯A	*d*(H⋯A) (Å)	∠DHA (°)	*d*(D⋯A) (Å)
N7–H7⋯ O9	1.936	155.18	2.759
N2–H2⋯O13	1.966	153.22	2.780
N5–H5⋯O9	1.933	174.46	2.810
C7–H7A⋯Cl4	2.784	139.52	3.560
C2–H2A⋯Cl5	2.781	136.39	3.530
N4–H4⋯O13	2.036	155.21	2.812

### 2.3 Powder X-ray diffraction data

The powder X-ray diffraction (PXRD) measurements were performed using a Panalytical MPD-PRO diffractometer equipped with a linear X'celerator detector and employing copper Kα radiation (Kα = 1.789 Å). Data were collected over a wide Bragg angle range of 5° to 100° to ensure comprehensive characterization of the crystalline material.

### 2.4 Spectroscopic measurements

Co-crystal formation was initially identified through vibrational analysis using both infrared (IR) and Raman spectroscopy. The analyses were conducted at room temperature using a PerkinElmer FT-IR Paragon 1000 PC spectrometer for IR measurements and a LABRAM HR 800 triple monochromator equipped with a 50× long-focus (LF) objective microscope for Raman spectroscopy, over the range of 500–4000 cm^−1^ for IR and 50–4000 cm^−1^ for Raman.

### 2.5 Optical measurements

The photoluminescence spectrum of PdzSb was recorded at room temperature using a PerkinElmer LS 55 spectrometer with an excitation wavelength of 350 nm. The optical absorption spectrum was recorded through direct transmission measurements using a Cary 5000 UV-Vis-NIR spectrophotometer.

### 2.6 NMR (^1^H and ^13^C) spectroscopy

Both the ^1^H and ^13^C NMR spectra were recorded on a Bruker AV400 Avance spectrometer operating at 400 MHz for ^1^H NMR and 100 MHz for ^13^C NMR. Chemical shifts are illustrated in parts per million (ppm) relative to tetramethylsilane (TMS) as the internal standard, with DMSO as the solvent.

### 2.7 Thermal analysis

The thermal properties of the PdzSb co-crystal were investigated by thermogravimetric analysis (TGA). Measurements were conducted using a PerkinElmer Pyris 6 TGA thermobalance at a heating rate of 10 °C min^−1^ under ambient air, over temperatures ranging from room temperature to 500 °C. Data acquisition and processing were performed with the PerkinElmer software.

### 2.8 Hirshfeld surface calculations

Hirshfeld surface analysis^41^ was applied to elucidate and spatially map the various non-covalent interactions,^42^ including the hydrogen bonding, π–π stacking, Pnictogen and halogen bonding, affording deep insights into molecular packing and intermolecular forces.

The analysis of these surfaces was carried out using the Crystal Explorer software^43^ based on electron-density distributions, using the CIF file as the input. In addition, 2D fingerprint plots^44^ were recorded to ensure a quantitative representation of the relative contributions of each type of intermolecular contact.

### 2.9 Computational details

A comprehensive theoretical investigation of the PdzSb co-crystal was carried out to elucidate its structural, electronic, and optical properties. Density functional theory (DFT) calculations were performed using the B3LYP exchange–correlation functional^45^ in conjunction with the LANL2DZ basis set,^46^ as implemented in Gaussian 09W.^47^ The molecular model employed in the calculations was extracted directly from the crystallographic CIF file, corresponding to the formula unit (identical to the asymmetric unit), and subsequently fully optimized in the gas phase without symmetry constraints. Vibrational frequency calculations were performed at the same level of theory to confirm the stability of the optimized structure, ensuring the absence of imaginary frequencies. The calculated vibrational frequencies were scaled using an appropriate scaling factor of 0.967 to correct the systematic overestimation inherent in the B3LYP/LANL2DZ level of theory. A comparison between the optimized geometry and the experimental X-ray structure revealed very good agreement, supporting the reliability of the adopted computational approach despite the gas-phase approximation. All subsequent electronic-structure analyses were carried out on the optimized geometry. This included frontier molecular orbital (HOMO–LUMO), electrostatic potential (MEP) mapping, electron localization function (ELF), localized orbital locator (LOL), and reduced density gradient (RDG) investigations. The latter analyses were performed using the Multiwfn program.^48^ It should be noted that the calculations were performed on an isolated molecular unit and, therefore, do not explicitly account for the crystal packing effects or the periodic boundary conditions. While this approximation may introduce minor discrepancies compared to the solid-state environment, it remains a widely adopted approach for obtaining qualitative and semi-quantitative insights into the intrinsic electronic properties of hybrid systems.

### 2.10 Determination of *in vitro* antibacterial activity by the agar well diffusion method

A volume of 100 µL of the bacterial suspension (10^6^ CFU mL^−1^) of each tested pathogenic strain was spread onto nutrient agar plates. Wells of approximately 10 mm in diameter were made using a sterile well cutter. Subsequently, 100 µL portions of 3,6-dichloropyridazine, PdzSb, and PdzBi solutions at a concentration of 100 mg mL^−1^ were added to the wells.^1^ The plates were incubated at 37 °C for 24 h. Antibacterial activity was evaluated by measuring the diameter of the inhibition zones formed around each well. Gentamycin (15 µg per well) was used as a positive control.^49^

### 2.11 Insecticidal bioassays

Insecticidal bioassays against *Ephestia kuehniella* larvae were performed using different concentrations of 3,6-dichloropyridazine, PdzSb and PdzBi.^1^ Ten second-instar larvae were exposed to serial concentrations of the tested compounds mixed with wheat semolina. Petri dishes were maintained under controlled conditions (24–28 °C, 16 h light/8 h dark photoperiod), and all experiments were conducted in triplicate. The LC_50_ (median lethal concentration), defined as the concentration of a substance causing 50% larval mortality, was estimated by probit analysis using R software.^50^

## 3. Results and discussion

### 3.1 Crystal studies

**Fig. 1:**
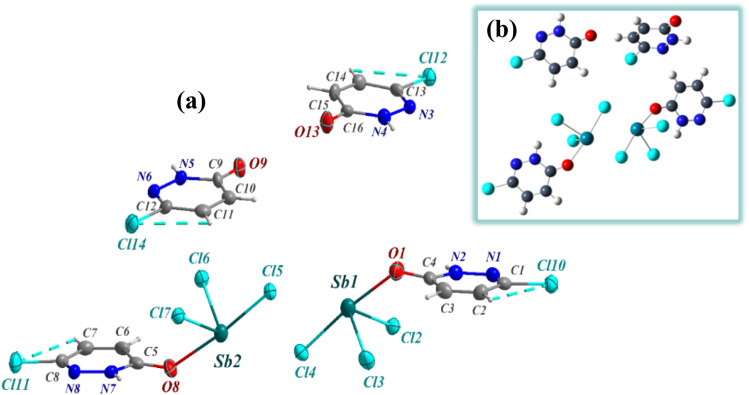
(a) Asymmetric unit and (b) theoretically optimized geometry of the PdzSb co-crystal. Note that the projections of both the free organic fragment and the antimony(iii) complex along different crystallographic directions were generated using the Diamond software.^51^ In fact, the main reason for the co-crystallization of the two antimony(iii) complexes and the two 6,3Pdz free ligands can be understood through their supramolecular interactions.

The composition and structural purity of the PdzSb co-crystal were assessed through energy-dispersive X-ray spectroscopy (EDS) combined with elemental mapping and powder X-ray diffraction (PXRD), indicating that the compounds are highly pure, as displayed in Fig. S1 and S2, respectively. The EDX analysis shows the presence of only N, O, Cl and Sb, without any contribution from extraneous elements, confirming the high chemical purity and compositional integrity of the PdzSb co-crystal with no evidence of significant contamination in the spectrum. On the other hand, the refined X-ray powder diffraction pattern of the compound recorded at room temperature exhibits excellent agreement with the pattern simulated from the single-crystal data. This concordance demonstrates that the title compound is a single-phase material, with no detectable impurities or secondary phases. Overall, this overlap further validates the structural model obtained from single-crystal X-ray diffraction. Single-crystal X-ray diffraction analysis reveals that the new compound, PdzSb, can be classified as a supramolecular compound that co-crystallized in the orthorhombic, with non-centrosymmetric space group *Fdd*2, exhibiting a peculiar assembly of components. The unit cell parameters are *a* = 17.5960 (4) Å, *b* = 28.4487 (7) Å, *c* = 24.7684 (6) Å, with *Z* = 16 and a unit-cell volume of *V* = 12 398.6 (5) Å^3^. Crystallographic data and more details on the crystal, data collection and refinement are summarized in Table 1. The asymmetric unit is built by four distinct neutral entities, including two free 6,3Pdz ligands and two discrete antimony-based complexes, each coordinated by three chloride and one protonated 6,3Pdz–O ligand through the oxygen atom, thereby balancing the overall charge of the complex, as indicated by the atom-numbering scheme (Fig. 1(a)). The corresponding optimized geometry of the formula unit for the newly synthesized compound is illustrated in Fig. 1(b).

**Fig. 2:**
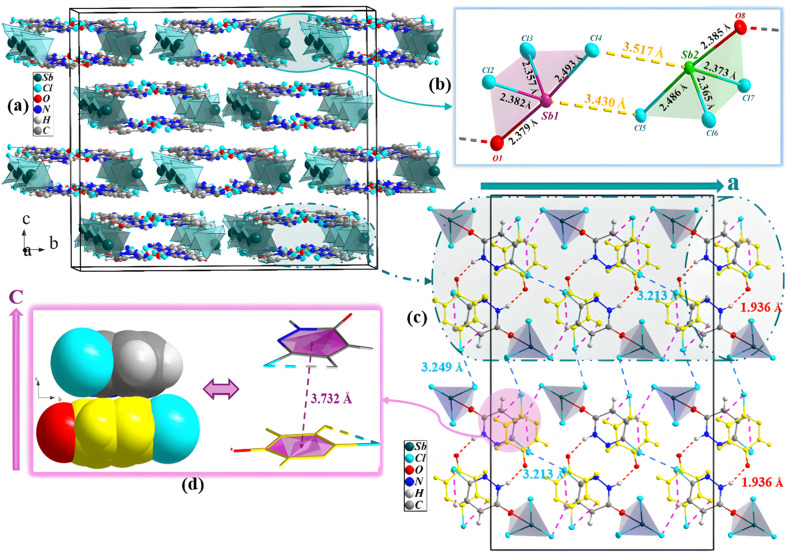
(a) Perspective view into the 3D network of the crystal structure of PdzSb and (b) variations in the Sb–Cl and Sb–O bond lengths within the polyhedron (dashed lines indicate Sb⋯Cl antiparallel dipole–dipole interactions). The (6,3Pdz–O) ligand has been omitted for the sake of clarity. (c) 1D tunnels formed by the components along the crystallographic *a* axis; halogen bonding Cl⋯Cl bonds are shown as blue-dotted lines, while the hydrogen bonds are shown as red and violet dotted lines. (d) Space-filling (left) and ball and stick (right) representations of the stacks of free 6,3Pdz ligands (yellow) and the ligand of the 6,3Pdz–O–Sb complex along the *c*-axis.

Fig. 2 reveals several insights into the crystalline architecture. Indeed, the self-assembly of the Sb complex and the 6,3Pdz free ligand (designed by yellow color) creates infinity one-dimensional tunnels along the *a* axis within the lattice that are interconnected *via* both halogen bonding Cl⋯Cl 3249 Å (dotted in blue lines) and Sb⋯Cl antiparallel dipole–dipole interactions (3.430(2)–3.517(3) A; dotted in orange lines), as displayed in Fig. 2(a)–(c).^6,21^ Besides, it is worth noting the presence of additional π–π stacking interactions, van der Waals contacts, and hydrogen bonds (N–H⋯O and C–H⋯Cl), which not only generate a dense and periodic three-dimensional network but also enhance the overall robustness of the crystal structure, as illustrated in Fig. 2(c) and (d). The geometric parameters of the H-bonds are summarized in Table 2. As for the Sb(iii) complex, each Sb atom exhibits a four-coordination geometry and conforms to an AX_4_E_1_ environment, consistent with a distorted seesaw geometry derived from a trigonal-bipyramidal electron arrangement.

**Fig. 3:**
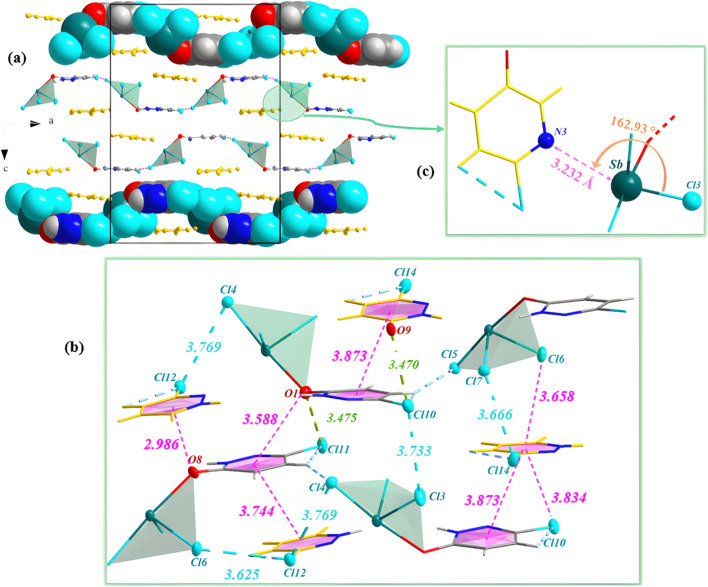
(a) Supramolecular chains along the *a* axis achieved through C–H⋯Cl hydrogen interactions (as indicated by the violet dotted lines). (b) Illustration of the Sb⋯N pnictogen bonds (dashed line). (c) Interconnection of chains *via* different types of π-stacking interactions (as indicated by the pink dotted lines); the halogen and hydrogen bonds in the compound's configuration are shown as green and blue dotted lines in the 3D network, respectively.

Owing to its higher electronegativity and stronger donor ability, the monodentate 6,3Pdz–O ligand coordinated through oxygen (Sb–O) occupies an axial position trans to one chloride ligand (Sb–Cl) with bond distances of of 2.379(5)–2.385(5) Å and 2.486 (18)–2.493(19) Å, respectively (longest values correspond to antiparallel dipole–dipole interactions), as shown in Fig. 2(b). The selected bond lengths and angles are given in Table S1. However, the two remaining chloride ligands reside in the equatorial plane with a distance ranging from 2.357 to 2.382 Å, whereas the third equatorial site is taken by the stereochemically active lone pair 5s^2^. This arrangement leads to a distinctly asymmetric distribution of the ligands around the Sb(iii) center, and the bond angles deviate from the ideal trigonal–bipyramidal structure due to steric constraints, pnictogens and hydrogen-bonding interactions.^6^ As depicted in Fig. 2(d), the sandwich motif comprises the aromatic π⋯π stacking between the 6,3Pdz–O of the Sb(iii) complex and the π electrons of the 6,3Pdz free ligand with a centroid–centroid distance of 3.732 Å along the 
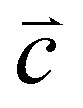
 axis. On the other hand, the projection in the [010] plane exhibits an infinity of chains formed by the Sb(iii) complex, which are interconnected *via* C–H⋯Cl hydrogen bonds and run parallel to the *a* axis, as shown in Fig. 3(a). However, the large voids generated by the 6,3Pdz–O–SbCl_3_ complex were occupied by the 6,3Pdz free ligand, which underscores the efficient packing achieved in the co-crystal structure. This atomic arrangement is similar to those found in the PdzBi co-crystal.^1^ Another view of the crystal packing along the *a* axis displays the antimony(iii) complex, which is sandwiched in between two infinity layers of both the 6,3Pdz free ligand and the 6,3Pdz–O complex ligand (Fig. S3). Fig. 3(b) provides a clearer view of different types of interactions in the unit cell. Both organic entities have a sandwich centrosymmetric structure, formed by Cl⋯π interactions between the chlorine atom and the π–electron cloud of an aromatic system of two adjacent 6,3Pdz–O ligands with a distance of 3.834 Å. Meanwhile, the supramolecular chains are formed through Cl⋯Cl halogen bonds with distances between 3.666 and 3.769 Å. Intermolecular π–π stacking interactions were also reported, with the distance between the centroids in the range of 3.744 (1)–3.873(4) Å. It can be noticed that the oxygen atoms from the 6,3Pdz–O ligand of the complex are not only involved in hydrogen bonding but also in the O⋯π interactions occurring between the oxygen atom and the centroid of adjacent aromatic rings, with a distance of 2.986 Å, as well as the O⋯Cl chalcogen interaction with a distance of 3.495 Å. Fig. 3(c) discloses a notable non-covalent interaction that plays a crucial role in the stability and organization of the crystal structure, namely, Sb⋯N pnictogen bonds (3.232 Å; with an angle of interaction of 162.93°), created between the adjacent 6,3Pdz–O–SbCl_3_ complexes and the unprotonated nitrogen of the free 6,3Pdz ligand, thus giving rise to two-dimensional networks parallel to the *ac* plane.^52^

Overall, the synergistic combination of hydrogen bonding and diverse non-covalent interactions offers a comprehensive explanation of the factors governing the coherence and stability of this co-crystal material. As previously reported in our earlier study,^1^ the neutral organic moiety, 6,3Pdz, in the co-crystal undergoes the same tautomeric conversion from pyridazin-3(2*H*)-one to pyridazin-3-ol already discussed extensively. Accordingly, only a concise summary is provided here. Fig. S2 provides a more detailed visualization supporting the DFT results, which confirms that the tautomer observed in the crystal structure is energetically favored due to carbonyl-group stabilization and intramolecular N–H⋯O hydrogen bonding, whereas steric repulsion between adjacent nitrogen lone pairs destabilizes the alternative form.^16,53^ These findings are fully consistent with the crystallographically determined structure and the tautomeric behavior reported in related studies.^1^

### 3.2 NMR (^1^H and ^13^C) spectroscopy

Insights into the molecular arrangement within the unit cell are further refined by the ^1^H and ^13^C NMR data presented in Fig. S4 and S5. The ^1^H NMR spectrum of the free ligand (6,3Pdz) shows a signal at 13.9 ppm, corresponding to the –NH proton,^16^ which is not detected in the complexes, reflecting deprotonation *via* keto–enol tautomerism and coordination through the C=O carbonyl oxygen.^54^ Moreover, the solid-state ^13^C NMR spectrum of the free 6,3Pdz displays a characteristic resonance at 160.14 ppm, attributable to the C=O carbon. Note that these findings exhibit the same trends as those identified in the PdzBi co-crystal.^1^
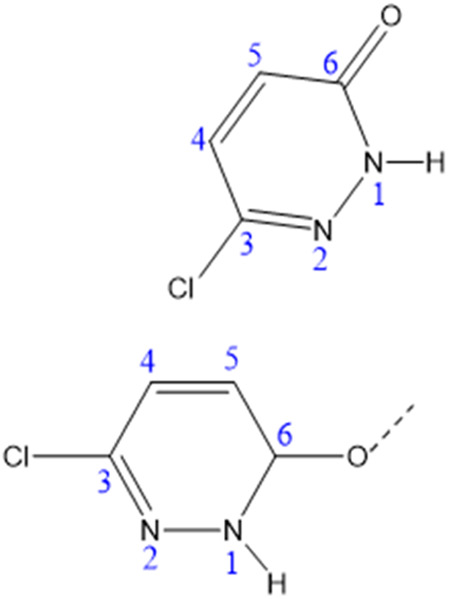


NMR ^1^H (400 MHz, DMSO-d_6_) [ppm] *δ* = [H5] 6.98 (d, 1H *J* = 9.8 Hz), [H4] 7.52 (d, 1H *J* = 9.8 Hz), [NH] 13.19.

NMR ^13^C (100 MHz, DMSO-d_6_) [ppm] *δ* = [C6] 160.13, [C3] 137.87, [C4] 135.24, [C5] 133.10.

### 3.3 Thermogravimetric analysis

The thermal stability and decomposition behavior of the PdzSb co-crystal were studied using thermogravimetric analysis (TGA) and differential thermogravimetric (DTG) analysis, Fig. S6.

Indeed, the TGA curve undergoes a well-defined three-step decomposition process, as further supported by the corresponding DTG profile. The initial drop from about 100% to roughly 57% (≈53.17% loss) between ∼150 °C and 230 °C is assigned to the complete release of the four organic ligands (4× C_4_H_3_N_2_ClO), both coordinated and uncoordinated, in excellent agreement with the calculated value (53.37%), as verified by the sharp peak in the DTG curve. This confirms that the organic components are the least thermally stable constituents of the co-crystal assembly. Note that the remaining inorganic residue corresponds to Sb_2_Cl_6_, originating from the two SbCl_3_ units of the initial structure. Following this step, a second smaller mass loss of 11.56%, observed between 300 and 500 °C, is attributed to the thermal conversion of Sb_2_Cl_6_ into the more stable oxychloride, Sb_2_O_2_Cl_2_. The relatively low DTG intensity displays a gradual process, consistent with the loss of four chlorine atoms as volatile chlorinated species (Cl_2_ and/or HCl), accompanied by the incorporation of atmospheric oxygen, yielding a theoretical net mass loss of 11.22%, in close agreement with the experimental value. Above 550 °C, no further significant decomposition occurs, and the final residue stabilizes at 35.27%, in good agreement with the calculated amount of Sb_2_O_2_Cl_2_ expected from the complete conversion of the inorganic precursor (35.41%). The nearly flat DTG baseline in this high-temperature region confirms that the Sb_2_O_2_Cl_2_ residue is the final thermally stable oxychloride phase formed under the conditions of the TGA experiment. Overall, the TGA/DTG data highlight that the compound exhibits excellent thermal stability up to approximately 200 °C, with no significant mass loss occurring in this temperature range. This stability reflects the strong intermolecular interactions within the crystal lattice, maintaining the structural integrity of both the organic and inorganic components under moderate thermal conditions.

### 3.4 Vibrational modes

**Fig. 4:**
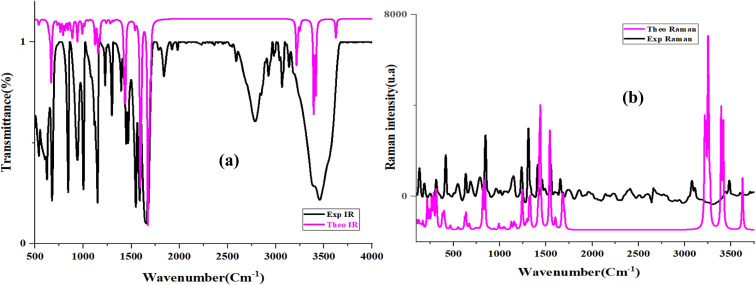
Experimental and theoretical IR (a) and Raman (b) spectra of PdzSb at room temperature.

The vibrational features of the PdzSb co-crystal were examined through IR and Raman spectroscopy studies (Fig. 4), with density functional theory (DFT) calculations (B3LYP/LanL2DZ) providing support for band assignments based on computed wavenumbers and previous vibrational studies.^1,16,55^

The IR spectrum displays the characteristic *ν*(N–H) stretching vibrations of the pyridazinone ring in the 3400–3200 cm^−1^ region, consistent with the presence of intramolecular hydrogen bonding (N–H⋯O), which stabilizes the ketonic form of the molecule. The asymmetric and symmetric *ν*(C–H) stretching modes are observed at 3381 cm^−1^. Furthermore, a notable shift of the *ν*(C–O) band to a higher frequency (1680 cm^−1^) is observed upon complexation, in contrast to the *ν*(C=O) stretching vibration of the 6,3Pdz free ligand, which appears around 1620 cm^−1^. The band at 1548 cm^−1^ is attributed to the *ν*(C=C) stretching modes. In addition, the absorptions at 1680, 1437 and 940 cm^−1^ correspond to the N–H in-plane bending, scissoring and out-of-plane bending modes, respectively. The region between 1298 and 1149 cm^−1^ includes *ν*(C–C), *ν*(C–Cl), *ν*(C–N) and *ν*(N–N) stretching vibrations, while the signals observed between 1118 and 847 cm^−1^ correspond to the out-of-plane bending, twisting motions and wagging modes of the (C–H) bonds. Further deformation modes involving the ring skeleton, such as *δ*(N–C–C) and *δ*(C–C), are identified near 670 cm^−1^. Importantly, the formation of the Sb–O bond upon complexation was supported by the appearance of a new *ν*(Sb–O) stretching vibration at 541 cm^−1^,^56^ thereby confirming the establishment of the 6,3Pdz–O–SbCl_3_ coordination environment.

The Raman spectrum of 6,3Pdz–O–SbCl_3_ (Fig. 4(b)) complements the IR data by revealing additional active modes, especially in the low-frequency region, associated with Sb–Cl and Sb–O bonds and lattice dynamics. The band located at 309 cm^−1^ is attributed to the stretching vibrations of Sb–O bonds, which indicate the coordination of oxygen atoms of the pyridazinon ring to Sb, while the signals at 255 and 190 cm^−1^ correspond to *ν*_as_(Sb–Cl) and *ν*_s_(Sb–Cl) bending modes.^54,57^ A distinct lattice mode is observed at 104 cm^−1^. The special Raman curve confirms the strong luminescence of the title compound, as shown in the literature.^58^

The Raman spectrum also exhibits an elevated background, attributed to the strong photoluminescence (PL) of the compound. Therefore, background correction was performed using polynomial baseline subtraction to clearly resolve the Raman bands.

To shed light on this unexpected observation and to gain deeper insights into the subtle balance of supramolecular interactions dictating the crystal packing, we performed a high-level density functional theory (DFT) study. The computed vibrational frequencies exhibit excellent concordance with the experimental observations, thereby enabling unambiguous mode assignments and supporting the structural robustness of the synthesized co-crystal. The tentative assignments of the experimental and theoretical vibrational frequencies of the title compound are mentioned in Table S2.

### 3.5 Hirshfeld surface analysis

**Fig. 5:**
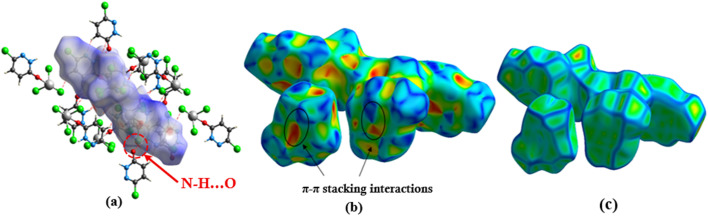
Hirshfeld surfaces of PdzSb: (a) 3D *d*_norm_ surface and (b) surface index and (c) curvedness.

To gain deeper insights into the intermolecular forces stabilizing the PdzSb co-crystal, a detailed Hirshfeld surface analysis was carried out using Crystal Explorer.^59^ The *d*_norm_ surface (Fig. 5(a)) displays several intense red regions, indicative of strong hydrogen-bonding interactions, such as N–H⋯O and C–H⋯Cl contacts. The shape index mapping (Fig. 5(b)) confirms the presence of π–π stacking interactions, evidenced by the characteristic adjacent red/orange and blue triangular regions. Similarly, the surface curvedness (Fig. 5(c)) exhibits extended flat regions in blue, further confirming the presence of π–π interactions within the crystal structure.

The two-dimensional fingerprint plots (Fig. S7) provide a quantitative breakdown of the intermolecular contacts contributing to the overall crystal packing. The Cl⋯H/H⋯Cl interactions constitute the major contribution,^1,11^ accounting for 27.2% of the total surface contacts, followed by the Cl⋯Cl (18%), O⋯H/H⋯O (10.5%) and N⋯H/H⋯N (9.6%) contacts, while the H⋯H interactions account for 6.2% of the surface and the N⋯Cl/Cl⋯N interactions account for 4.1%. Other intermolecular contacts contribute less than 4% to the overall surface interactions, as shown in Fig. S7.

Void analysis was carried out to characterize the presence, size, and distribution of the unoccupied regions within the crystal packing of the PdzSb co-crystal. Using the procrystal electron density isosurface method implemented in Crystal Explorer,^59^ the voids were quantified by summing the spherical atomic electron densities centered on each nuclear position, allowing the accurate mapping of the space within the unit cell.^60^ As shown in Fig. S8, the total void volume is 1243.60 Å^3^, relative to a unit cell volume of 12 398.6(5) Å^3^, corresponding to a void fraction of approximately 10.03%. This relatively low proportion of unoccupied space reflects a highly efficient compact packing arrangement, with no evidence of large, interconnected cavities. Such a structural density typically corresponds to enhanced mechanical stability, limited molecular mobility, and potentially reduced solubility, all of which contribute to the robustness of the crystalline material.

### 3.6 Electrostatic and non-covalent interaction-based study

A comprehensive analysis combining molecular electrostatic potential (MEP), non-covalent interaction analysis based on the reduced density gradient (NCI-RDG), electron localization function (ELF), and localized orbital locator (LOL) was carried out to gain deep insights into the electronic structure and intermolecular interactions within the co-crystal material. In turn, the MEP surface delineates the electrophilic and nucleophilic regions, thereby identifying potential interaction sites, whereas the NCI-RDG analysis reveals weak interactions, including hydrogen bonding and van der Waals forces that govern the supramolecular architecture of the material. Meanwhile, ELF and LOL investigations provide complementary views of the electron localization and orbital features relevant to bonding and reactivity. These advanced approaches elucidate the charge distribution, non-covalent bonding features, and electron localization, which are crucial for understanding the stability and functional properties of the PdzSb system.

#### 3.6.1. Molecular electrostatic potential analysis

**Fig. 6:**
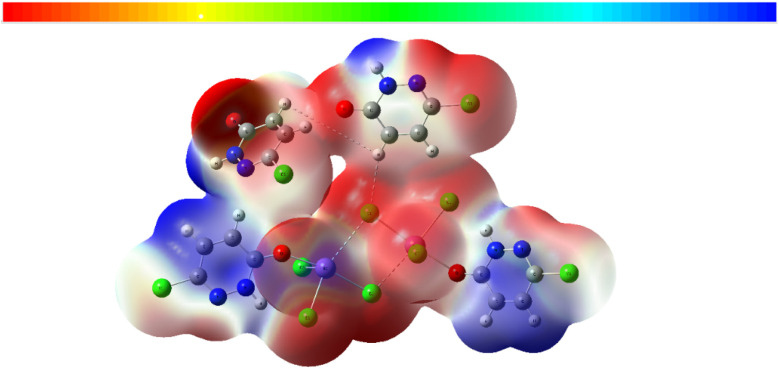
Molecular electrostatic potential (MEP) map of the PdzSb co-crystal. The color ranges from red (most negative potential) to blue (most positive potential), with values given in arbitrary units (a.u.).

The molecular electrostatic potential (MEP) surface of the co-crystal provides valuable insights into the distribution of electronic charges and the regions susceptible to electrophilic or nucleophilic interactions (Fig. 6). The MEP map displays a heterogeneous charge distribution, with intense red zones corresponding to regions of high electron density (negative potential), mainly localized around the electronegative chlorine atoms and oxygen atoms of the organic ligand. These negatively charged regions represent the most favorable sites for electrophilic attack, and they correlate well with the experimentally observed involvement of Cl and O atoms in noncovalent interactions, such as C–H⋯Cl, N–H⋯O, Sb⋯Cl and weak Cl⋯Cl contacts.^23^ Nevertheless, the blue regions on the MEP surface indicate areas of low electron density (positive potential), predominantly associated with the hydrogen atoms of the organic units and the Sb(iii) centers, which serve as potential nucleophilic attack sites. These electropositive regions are consistent with the hydrogen-bond donor sites observed in the crystal packing.

Overall, the MEP analysis confirms that the co-crystal's stability is ensured by a combination of electrostatic interactions, hydrogen and pnictogen bonding and halogen-based contacts, in agreement with the Hirshfeld and RDG results. These findings provide a deeper understanding of the compound's reactivity and physicochemical properties, particularly its tendency to form noncovalent interactions. To further address the limitations of the co-crystal MEP representation, the molecular electrostatic potential surfaces of the individual molecular components were additionally computed and are presented in Fig. S9. These surfaces provide a more localized and reliable description of the charge distribution, enabling a clearer identification of the actual regions involved in the intermolecular interactions. The individual MEP maps show that the most negative electrostatic potential is mainly localized around electronegative atoms, particularly Cl and O, confirming their role as preferential sites for electrophilic interactions. In contrast, the positive potential regions are predominantly distributed over hydrogen atoms and around the Sb(iii) centers, indicating their involvement as nucleophilic interaction sites. These results are consistent with the experimentally observed noncovalent interactions and provide a more accurate representation of the interaction-prone regions compared to the co-crystal MEP surface.

#### 3.6.2. ELF and LOL analyses

**Fig. 7:**
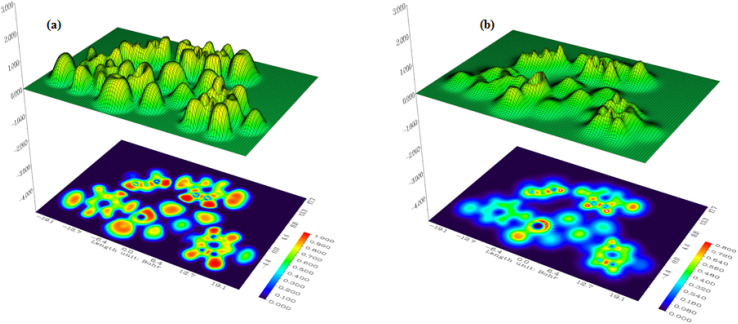
Electron localization function (ELF) maps (a) and (b) localized orbital locator (LOL) maps of the PdzSb co-crystal.

The electronic distribution within the co-crystal was further explored by electron localization function (ELF) and localized orbital locator (LOL) analyses,^1,61,62^ as presented in Fig. 7.

The ELF map (Fig. 7(a)) highlights strong electron localization around the O, N, and Cl heteroatoms, reflecting their high electronegativity and the presence of well-defined lone-pair regions. These localized electron domains are consistent with their participation in the key intermolecular contacts identified in the crystal, particularly the N–H⋯O and C–H⋯Cl hydrogen-bond-type interactions. In contrast, the Sb–Cl environment exhibits broader and less localized ELF features, in line with the mixed ionic–covalent nature of Sb–Cl bonding. Similarly, the LOL maps (Fig. 7(b)) provide complementary insights into the delocalization of electrons across the molecular framework. High LOL values are concentrated around the heteroatoms and within the σ-bonding regions, confirming well-defined covalent interactions. Notably, the organic aromatic rings exhibit regions of enhanced LOL delocalization, supporting the presence of π–electron clouds that can engage in π–π stacking interactions. Together, the ELF and LOL visualizations highlight the dominance of localized lone pairs and π–electron delocalization in governing the stability and supramolecular organization of the co-crystal.

#### 3.6.3. Reduced density gradient (RDG) analysis

**Fig. 8:**
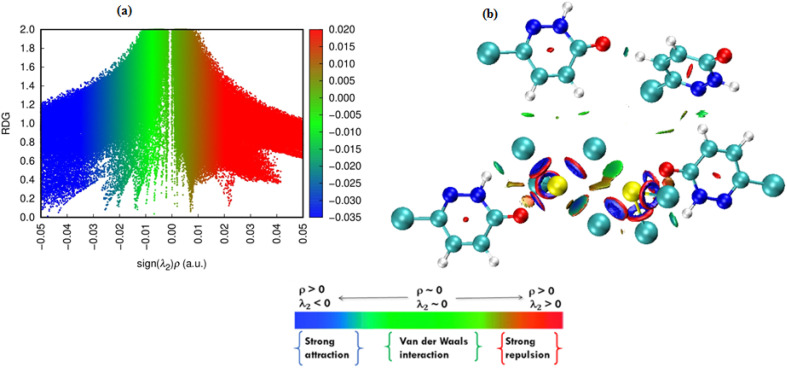
Reduced density gradient scatter plot (a) and color-filled isosurface (b) of the PdzSb co-crystal illustrating the non-bonded interactions.

The non-covalent interactions governing the supramolecular stability of the PdzSb co-crystal were further examined through the reduced density gradient (RDG) approach with Multiwfn and visualized *via* the VMD program,^63^ as presented in Fig. 8. The RDG scatter plot (Fig. 8(a)), which represents RDG values as a function of sign(*λ*_2_)*ρ*, clearly distinguishes the different types of intermolecular contacts.

The presence of pronounced spikes in the negative sign(*λ*_2_)*ρ* region reflects attractive interactions, mainly attributed to N–H⋯O and C–H⋯Cl hydrogen-bond-type contacts previously identified in the structural study. The broad green domain around sign(*λ*_2_)*ρ* ≈ 0 corresponds to dispersive van der Waals forces, which contribute significantly to the packing cohesion, especially between adjacent organic fragments. Conversely, the positive sign(*λ*_2_)*ρ* zone, represented in red, indicates steric repulsion. The color-filled RDG isosurface (Fig. 8(b)) provides a spatial representation of these interactions, clearly highlighting the attractive hydrogen-bonding zones in blue and light green, as well as the weak dispersive contacts in green. Notably, the elliptic red plate located at the center of the aromatic nucleus^64^ reflects the presence of π–π stacking interactions and illustrates the steric effects arising from close face-to-face overlap of aromatic rings. Together, the RDG scatter plot and isosurface confirm that the stability of the PdzSb co-crystal arises from a combination of hydrogen bonds, halogen–halogen contacts, π–π stacking interactions and van der Waals interactions, which collectively shape its supramolecular architecture.

In addition, special attention should be given to the role of the stereochemically active lone pair of the Sb(iii) center in shaping the observed RDG features. The presence of this lone pair induces an anisotropic distribution of electron density around the antimony atom, which contributes to the formation of localized noncovalent interaction regions in the RDG isosurfaces. These interaction domains, although weaker compared to classical hydrogen bonding, play an important role in directing the supramolecular assembly. Therefore, the RDG analysis further confirms that the lone pair is not only responsible for the distorted seesaw geometry but also actively participates in governing the intermolecular interaction landscape of the crystal.

### 3.7 UV-vis and photoluminescence properties

**Fig. 9:**
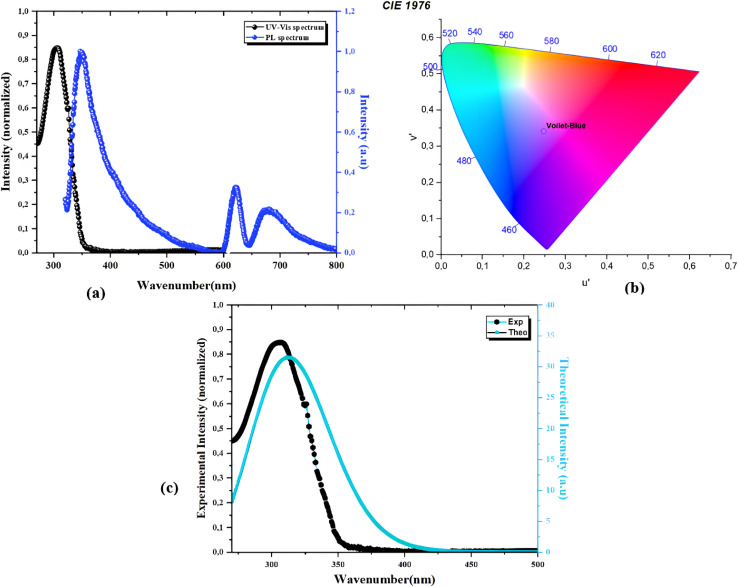
(a) UV-vis spectrum (black) and photoluminescence profile (blue), (b) chromaticity diagram and (c) experimental intensity (black) and theoretical intensity (cyan) of the PdzSb compound.

The optical properties of the synthesized Sb(iii)-based co-crystal were investigated through UV-Vis absorption and photoluminescence spectroscopy, supported by chromaticity analysis and density functional theory (DFT) calculations, providing a detailed understanding of the structure–property relationship. As shown in Fig. 9(a), The UV-Vis absorption spectrum exhibits an intense and well-defined band in the ultraviolet region, around 320 nm, which is attributed to the π → π* and n → π* electronic transitions mainly localized on the organic ligand. The narrowness and intensity of this band suggest a highly localized electronic transition, influenced by coordination to the Sb(iii) centers. Upon UV excitation, the photoluminescence (PL) spectrum reveals a dominant emission peak around 380–400 nm in the violet-blue region, indicating that the electronic de-excitation is predominantly localized on the ligand. Additionally, two weaker emission bands are observed in the visible region (620–700 nm), suggesting the presence of lower-energy emissive states associated with metal–ligand interactions or supramolecular effects within the co-crystal lattice. Therefore, the optical behavior demonstrates that the co-crystal exhibits strong violet-blue fluorescence with secondary red emissions, highlighting its potential for applications in optoelectronic devices, fluorescence-based sensing, or other photonic materials.^25^ The emission characteristics were further analyzed using the CIE 1976 chromaticity diagram (*u*′, *v′*). The calculated chromaticity coordinates are approximately (0.165, 0.175) in the violet-blue region of the color space, confirming the spectroscopic observations and indicating that the emission is dominated by high-energy electronic transitions, as seen in the chromaticity diagram in Fig. 9(b).

This chromatic behavior highlights the potential of the material for applications requiring short-wavelength emission. A strong correlation is observed between the experimental UV-Vis and photoluminescence data of the PdzSb co-crystal and the corresponding theoretical results, whereas the presence of minor discrepancies can be explained by the approximations of the computational model and the absence of certain experimental effects, such as solvation and intermolecular interactions (Fig. 9(c)).

**Fig. 10:**
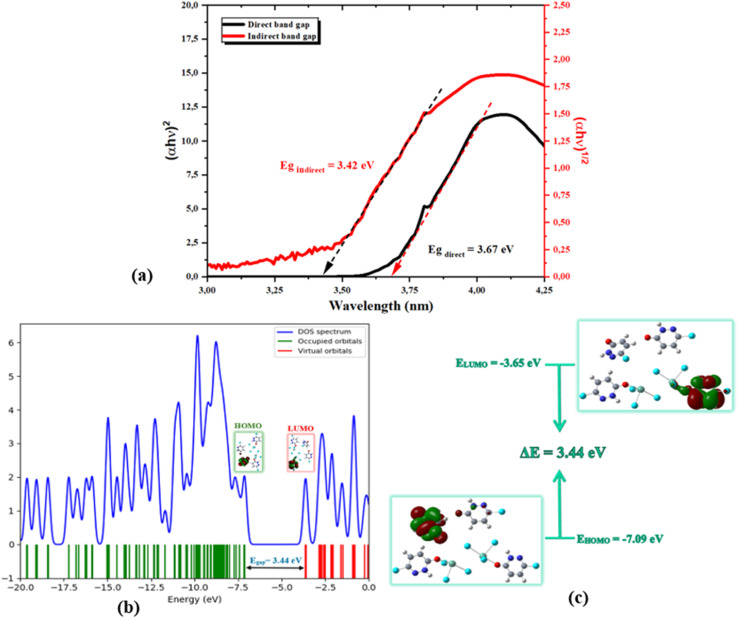
(a) Evolution of (*αhν*)^1/2^ and (*αhν*)^2^ with energy *hν* of the studied PdzSb co-crystal. (b) Density of state (DOS) spectrum and (c) HOMO and LUMO molecular orbitals of the PdzSb compound.

The optical band gap values were determined from Tauc plots,^65^ derived from the UV-Vis absorption data. The co-crystal exhibits both direct and indirect transitions, with estimated band gap energies of approximately 3.67 eV for the direct transition and 3.42 eV for the indirect one, as determined from the extrapolation of the linear regions of the (*αhν*)^1/2^ and (*αhν*)^2^ plots (Fig. 10(a)). In fact, the coexistence of both direct and indirect transitions suggests a complex electronic structure, where phonon-assisted processes may contribute to optical absorption near the absorption edge. Moreover, the close proximity of these values suggests contributions from both transition types, probably arising from the metal–ligand orbital hybridization and structural distortion associated with the stereochemically active lone pair of the Sb(iii) center. Thus, these wide band gap values are consistent with the strong UV absorption and blue-violet emission observed experimentally, confirming the semiconducting nature of the material and its suitability for optoelectronic applications.^25^ A comprehensive analysis of the electronic structure was conducted through the density of states (DOS) and frontier molecular orbital calculations. The calculated total density of states (DOS) reveals the electronic structure of the co-crystal. The valence band is predominantly composed of occupied orbitals, whereas the conduction band consists mainly of virtual orbitals and provides additional confirmation of the indirect band gap nature (Fig. 10(b)). The DOS peaks suggest significant contributions from both Sb–Cl and organic ligand orbitals, reflecting the hybrid nature of the co-crystal. Nevertheless, the analysis of the frontier molecular orbitals shows that the highest occupied molecular orbital (HOMO) is mainly localized on the organic ligand, indicating that the valence band is mainly governed by ligand-centered electronic states. In contrast, the lowest unoccupied molecular orbital (LUMO) displays contributions from both the ligand and the Sb(iii) center, suggesting partial metal–ligand hybridization in the conduction band.

**Fig. 11:**
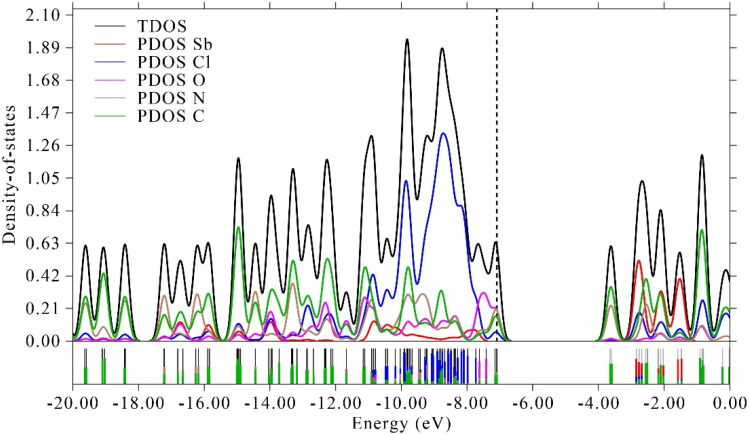
Total and partial density of states (TDOS and PDOS) of PdzSb.

This spatial distribution supports the assignment of the optical transitions as primarily ligand-centered with a metal-modulated character. The calculated HOMO and LUMO energy levels are approximately −7.09 eV and −3.65 eV, respectively, resulting in an energy gap (Δ*E*) of about 3.44 eV. This value is in excellent agreement with the experimentally determined optical band gaps, validating the reliability of the theoretical model (Fig. 10(c)). Meanwhile, the electronic structure and the nature of the orbital contributions to the valence band were further elucidated by total and partial density of states (TDOS and PDOS) analyses (Fig. 11).

The TDOS shows that the valence band is primarily composed of major contributions from Cl and C atoms and minor contributions from N and O atoms, whereas the Sb atoms contribute mainly to the deeper energy region, indicating the presence of hybridized bonding between the metal center and the heteroatoms of the coordinated organic ligands. However, the conduction band includes contributions from both the ligand framework and the Sb 5p orbitals. This orbital distribution confirms that the optical transitions are primarily ligand-centered, with a noticeable metal–ligand hybridization that plays a crucial role in modulating the electronic and photophysical properties. The lack of electronic states at the Fermi level (set at 0 eV) evidences a well-defined band gap, consistent with semiconductor behavior. The uncoordinated organic molecule introduces additional states in the vicinity of the Fermi level, indicating that its role is mainly associated with crystal packing and intermolecular interactions rather than charge transport. Consequently, the PDOS analysis supports the description of the material as a molecular co-crystal with insulating or semiconducting properties.

### 3.8 Biological studies

#### 3.8.1. Antibacterial activity

**Fig. 12:**
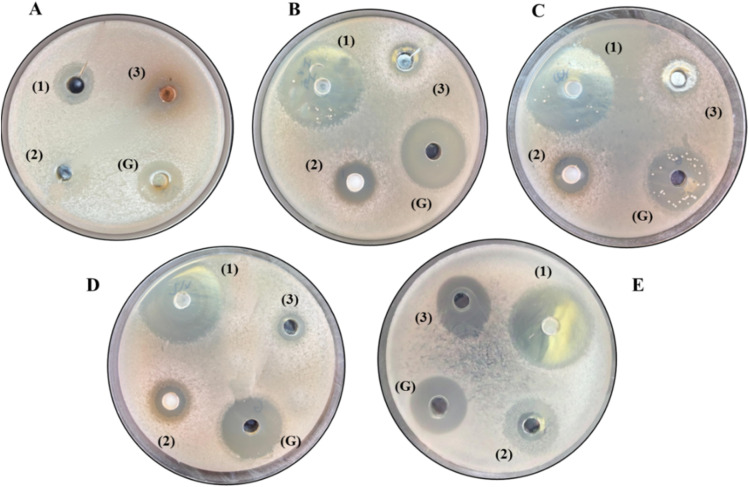
Inhibition zone of PdzSb (1), PdzBi (2) and 3,6-dichloropyridazine (3) against bacteria: (A) *Salmonella typhimurium*; (B) *Pseudomonas aeruginosa*; (C) *Bacillus subtilis*; (D) *Staphylococcus aureus*; (E) *Enterococcus faecalis*; and (G): gentamycin.

The antimicrobial activity of 3,6-dichloropyridazine, PdzSb and PdzBi^1^ was evaluated using the agar well diffusion method against two Gram-positive bacteria (*Staphylococcus aureus* and *Bacillus subtilis*) and three Gram-negative pathogenic bacteria (*Pseudomonas aeruginosa*, *Enterococcus faecalis*, and *Salmonella typhimurium*) (Fig. 12). The inhibition zone diameters are presented in Table 3. In summary, *Salmonella typhimurium* is a major foodborne pathogen; *Pseudomonas aeruginosa* can cause pneumonia, chronic obstructive pulmonary disease, and complications in cystic fibrosis. *Enterococcus faecalis* is associated with inflammatory diseases and severe nosocomial infections. *Bacillus cereus* is recognized as an etiological agent of eye and systemic infections, and *Staphylococcus aureus* can lead to a wide range of diseases, from moderate skin infections to life-threatening pneumonia and sepsis.^66–70^ Previous studies showed that PdzBi co-crystal exhibited similar activity to 3,6-dichloropyridazine against *Salmonella typhimurium* and *Staphylococcus aureus*, indicating no effect of BiCl_3_ addition, while enhanced activity was observed against *Pseudomonas aeruginosa* and *Enterococcus faecalis*.^1^ In the present study, we compared the antibacterial activities of PdzSb and PdzBi co-crystals against pathogenic bacteria in order to evaluate the effect of antimony and bismuth addition to 3,6-dichloropyridazine against these pathogens. As shown in Table 1, the PdzSb co-crystal showed the highest inhibition zones against all tested bacteria, significantly exceeding those of 3,6-dichloropyridazine and gentamicin antibiotics.

**Table 3:** Inhibition zones (mm) of PdzSb, PdzBi and 3, 6-dichloropyridazine against pathogenic bacteria

Bacteria	Inhibition zones (mm)			
	3,6-Dichloropyridazin	PdzSb	PdzBi	Gentamycin antibiotic
*Salmonella typhimurium*	12	19	11	17
*Pseudomonas aeruginosa*	11	35	18	23
*Bacillus subtilis*	18	34	14	25
*Staphylococcus aureus*	12	32	12	23
*Enterococcus faecalis*	11	30	20	20

This improvement was especially pronounced against *Pseudomonas aeruginosa* (IZ = 35 mm), *Bacillus subtilis* (IZ = 34 mm), and *Staphylococcus aureus* (IZ = 32 mm), suggesting that antimony incorporation plays a key role in enhancing the compound's antibacterial efficacy.^12,71^ Notably, PdzSb consistently outperformed PdzBi in terms of inhibition zones across all tested bacteria. This observation underscores that antimony enhances antibacterial activity more effectively than bismuth, highlighting PdzSb as the more potent 3,6-dichloropyridazine-based derivative.

#### 3.8.2. Larvicidal activity

Despite increasing attention toward eco-friendly and biological control strategies, chemical pesticides remain among the most widely used and effective tools in larval control programs. Conventional synthetic insecticides, including organophosphates, pyrethroids, and other neurotoxic compounds, have been extensively applied for decades owing to their rapid action and high mortality rates in larval populations.^72^ In fact, certain heterocyclic compounds, such as 3,6-dichloropyridazine, are not directly employed as commercial pesticides due to their insufficient intrinsic pesticidal activity and limited target selectivity. Instead, they are primarily utilized as versatile chemical intermediates in agrochemical synthesis.^23,73^ To overcome these limitations, structural modification through the introduction of functional groups, such as amines, thiols, heterocycles, or phenoxy substituents, has been widely explored. Such substitutions can significantly alter the biological profile of pyridazine-based molecules, conferring enhanced herbicidal, fungicidal, or insecticidal properties.^74^ In addition, several studies have demonstrated that bismuth-containing compounds exhibit significant larvicidal activity, particularly against mosquito vectors. For instance, bismuth(iii) dithiocarbamate complexes exhibit high toxicity toward *Aedes aegypti* larvae, with LC_50_ and LC_90_ values of 7.53 and 14.43 ppm, respectively.^75^ Furthermore, bismuth-containing nanomaterials, including Bi–Ag oxide nanocomposites and bismuth oxide nanoparticles, have been reported to induce significant larval mortality in *Aedes* and *Culex* species, likely through oxidative and membrane-disruptive mechanisms.^76^ In contrast, antimony compounds have been predominantly investigated for pharmacological applications, such as antiprotozoal activity, particularly in the treatment of leishmaniasis, and there is limited evidence of their larvicidal or insecticidal potential.^77^ In view of the paucity of reports concerning the larvicidal activity of antimony and bismuth compounds, the present study aimed to combine 3,6-dichloropyridazine with both bismuth and antimony centers to evaluate their insecticidal activities against the Mediterranean flour moth, *Ephestia kuehniella*. This larva is a stored-product pest that feeds on flour and grain, causing important economic losses.^78^ The LC_50_ values for 3,6-dichloropyridazine, PdzSb, and PdzBi were approximately 77.47 ± 3.76, 111.42 ± 11.14, and 143.67 ± 14.75 mg mL^−1^, respectively. These results indicate that all three compounds exhibit measurable insecticidal activity against *E. kuehniella* larvae. Interestingly, the antimony-containing derivative showed higher efficacy than its bismuth counterpart when combined with the 3,6-dichloropyridazine scaffold. Overall, these findings highlight antimony as a promising synergistic element capable of enhancing the insecticidal potential of pyridazine-based compounds against stored-product pests, offering valuable insights for the development of more effective larvicidal agents.

## 4. Conclusion

In this study, we described the synthesis, characterisation, and biological activity of a novel antimony(iii)-based co-crystal. Structural analysis confirmed a well-organized crystalline framework with strong coordination between the antimony centers and the oxygen atoms of the 6,3Pdz–O ligands. Importantly, the Sb(iii) center adopts a seesaw geometry, reflecting the stereochemical influence of the lone pair and constituting a noteworthy feature of the coordination environment. A key novelty of this work lies in establishing a clear correlation between the stereochemically active lone pair and the resulting structural organization, which directly governs the observed physicochemical properties. Surprisingly, in the literature, this compound has been infrequently reported to exhibit novel structural topologies and versatile coordination modes that expand the current understanding of Sb(iii) coordination chemistry. The cohesion of crystal packing was achieved by a synergistic network of hydrogen, halogen, and pnictogen bonds, along with π–π stacking interactions, as evidenced by Hirshfeld surface analysis. Complementary theoretical investigations, including ELF, LOL, and RDG analyses, revealed the coexistence of covalent and noncovalent interactions, cooperatively ensuring the compound's structural robustness. Additionally, the geometric parameters, vibrational frequencies, UV-vis spectrum, and HOMO–LUMO analysis for geometry optimization calculated at the B3LYP/LANL2DZ level presented good agreement with experimental data. The optical band gap estimated from UV-Vis spectroscopy was 3.42 eV for a direct allowed transition, confirmed by DFT calculations, thereby establishing the material as a promising candidate for optoelectronic applications. Importantly, biological evaluations highlighted the potent antibacterial activity of the material against a range of clinically relevant human pathogens, with antimony incorporation significantly enhancing bioactivity compared to the analogous bismuth-based derivatives. This behavior suggests that the coordination of SbCl_3_ is a good strategy for the development of new antibiotics derived from 3.6-dichloropyridazine. Overall, the results demonstrate that the physicochemical and biological properties observed in this co-crystal are strongly governed by the lone-pair-driven structural features, highlighting the importance of evaluating structure–property relationships rather than focusing solely on the optimization of a single application.

The summary of the development of antimony and bismuth compounds as antibacterial agents clearly indicates the potential for these elements to augment their therapeutic roles. Nevertheless, preliminary tests suggest the co-crystal's promising potential in biological control applications, expanding its relevance beyond conventional antimicrobial use. Overall, these findings reveal that antimony compounds hold great promise and, therefore, deserve further research. They also highlight the dual functionality of this co-crystal, which combines advanced material properties with significant bioactive performance, positioning it as a versatile candidate for future applications in antimicrobial therapies and sustainable biocontrol strategies.

## Conflicts of interest

The authors declare that they have no known competing financial interests or personal relationships that could have appeared to influence the work reported in this paper.

## Supplementary Material

RA-016-D6RA01859A-s001

RA-016-D6RA01859A-s002

## Data Availability

The raw/processed data required to reproduce these findings are available and can be sent if requested. CCDC 2449627 contains the supplementary crystallographic data for this paper.^79^ Supplementary information (SI) is available. See DOI: https://doi.org/10.1039/d6ra01859a.
