# Supplementary material for: Unveiling the supramolecular assembly of a novel antimony(iii)-based co-crystal: integrating multi-spectroscopic analyses, DFT calculations, and advanced biological evaluations
Source: RSC Adv. 2026 May 13;16(28):25251–68. doi: 10.1039/d6ra01859a (PMC13169108; doi:10.1039/d6ra01859a)
Supplement: RA-016-D6RA01859A-s001 [file RA-016-D6RA01859A-s001.pdf]

This journal is © The Royal Society of Chemistry 2026

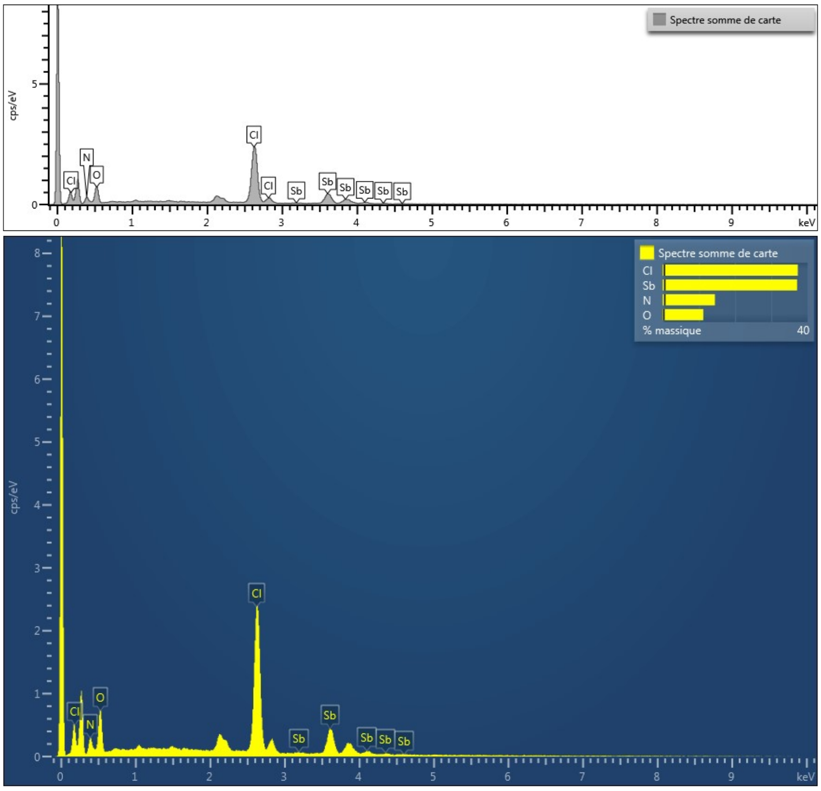

Élément	% atomique
N	33,33
O	22,68
Cl	34,11
Sb	9,88
Total:	100

PdzSb 10 kV x1000

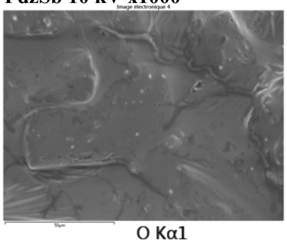

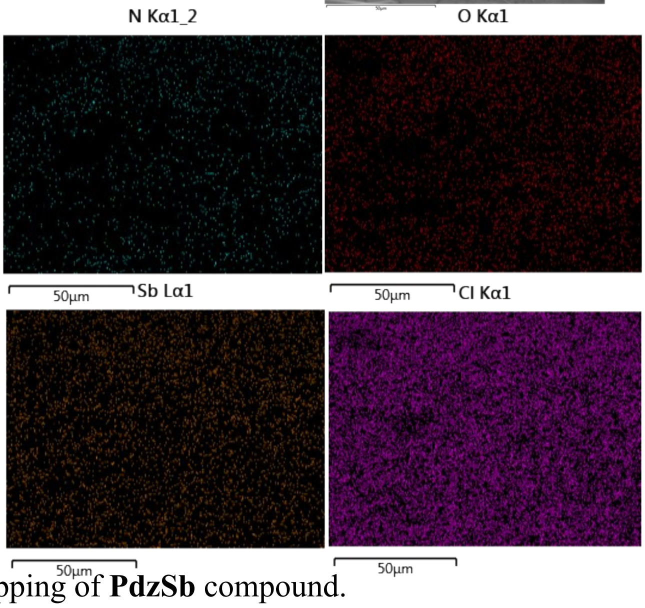

Fig S1.EDS spectrum and elemental mapping of **PdzSb** compound.

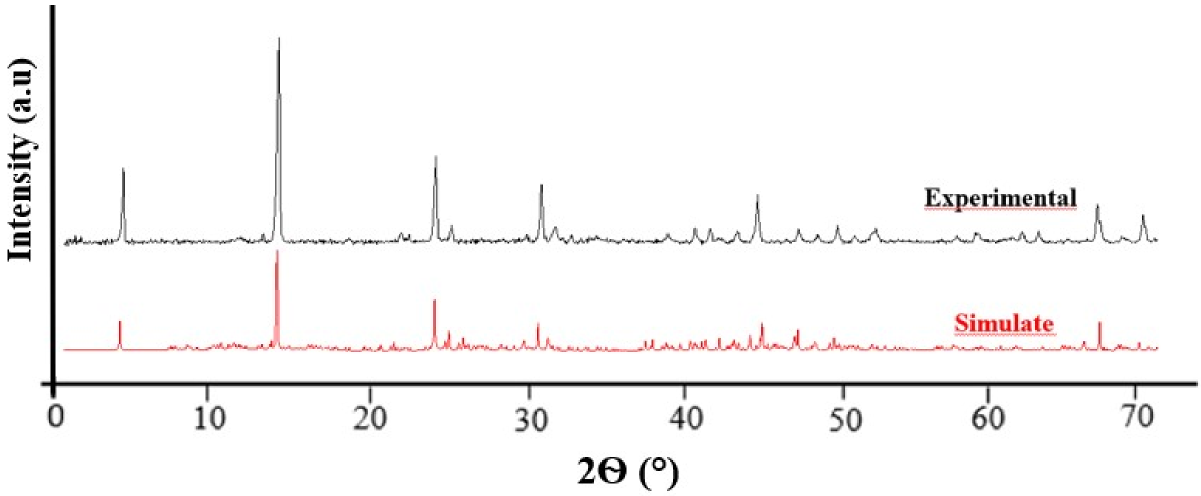

Fig. S2. PXRD pattern with experimental (black) and simulated (red) data of **PdzSb** compound.

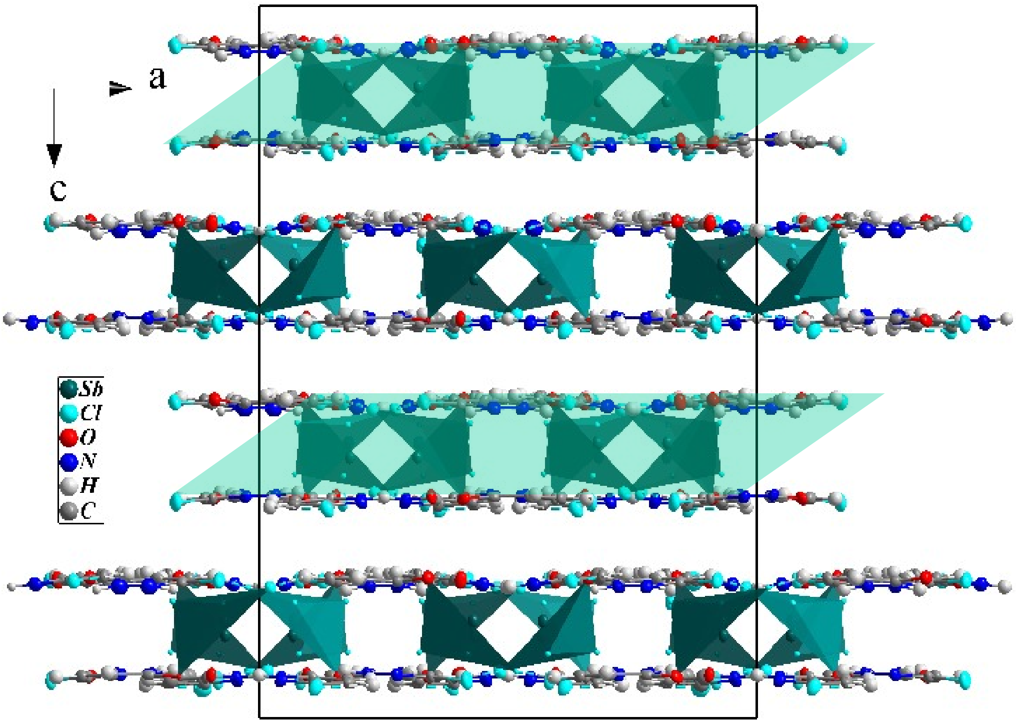

Fig. S3. Other projection of the **PdzSb** co-crystal in (ac) plan.

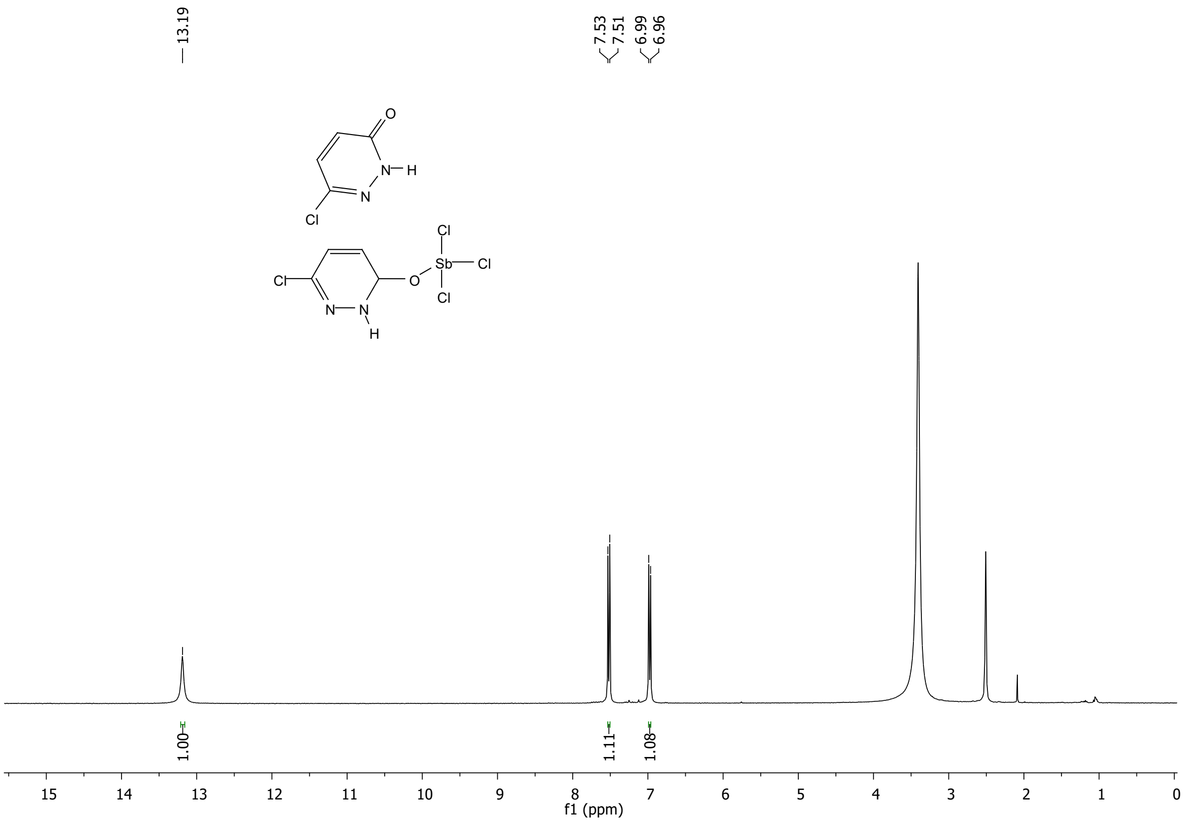

Fig. S4. NMR ^{1}H (400 MHz, DMSO-d_6)

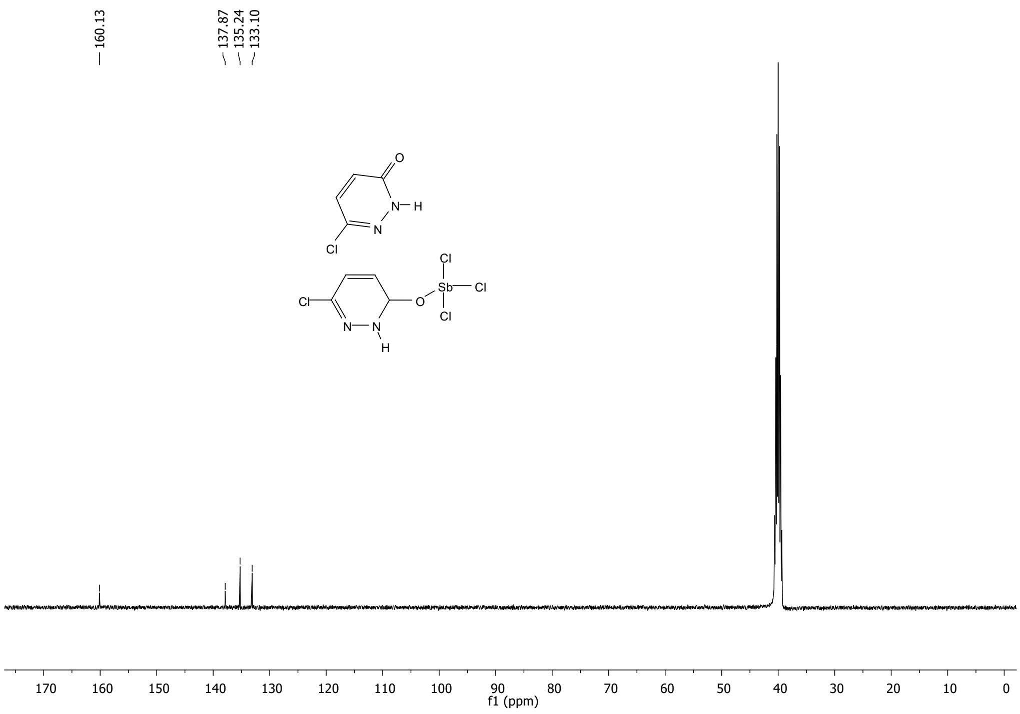

Fig. S5. NMR ^{13}C (100 MHz, DMSO-d_6)

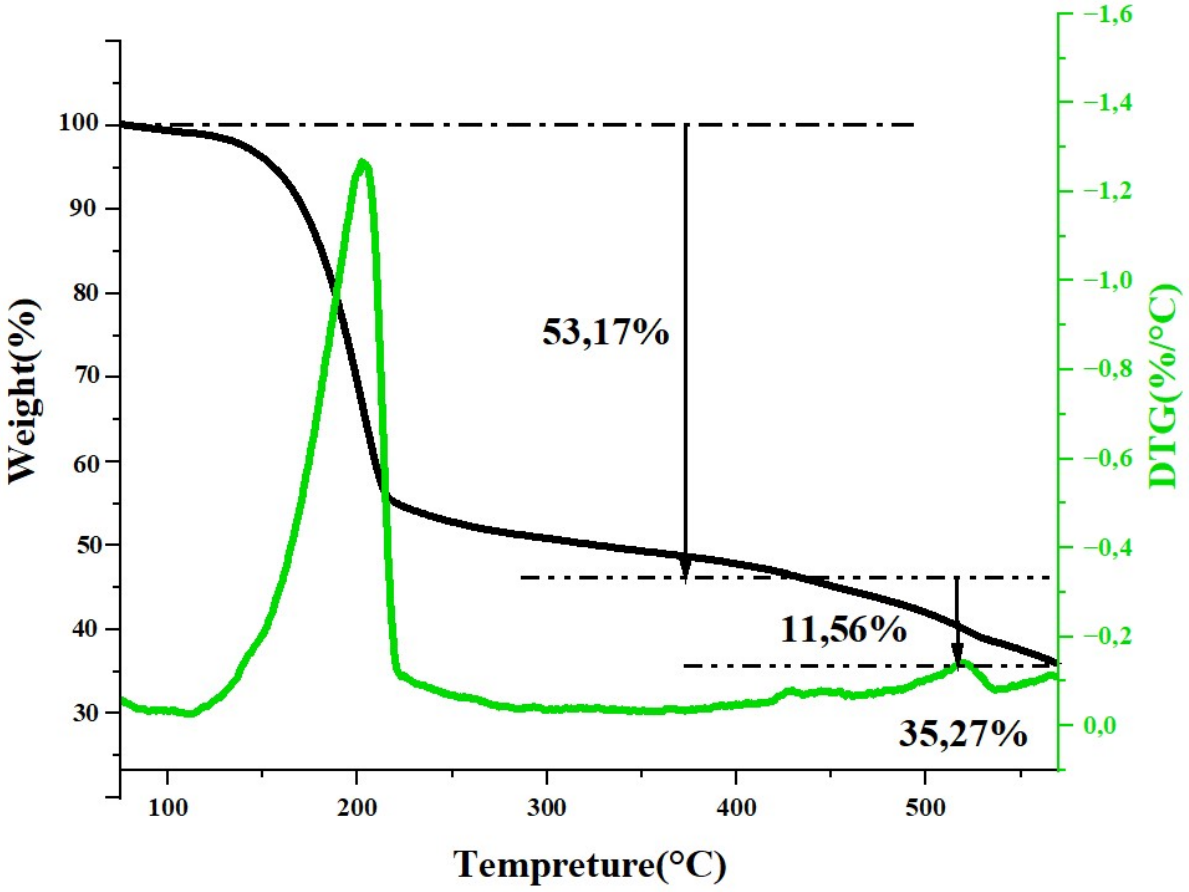

Fig. S6. TGA-DTG analysis plots of **PdzSb** cocrystal.

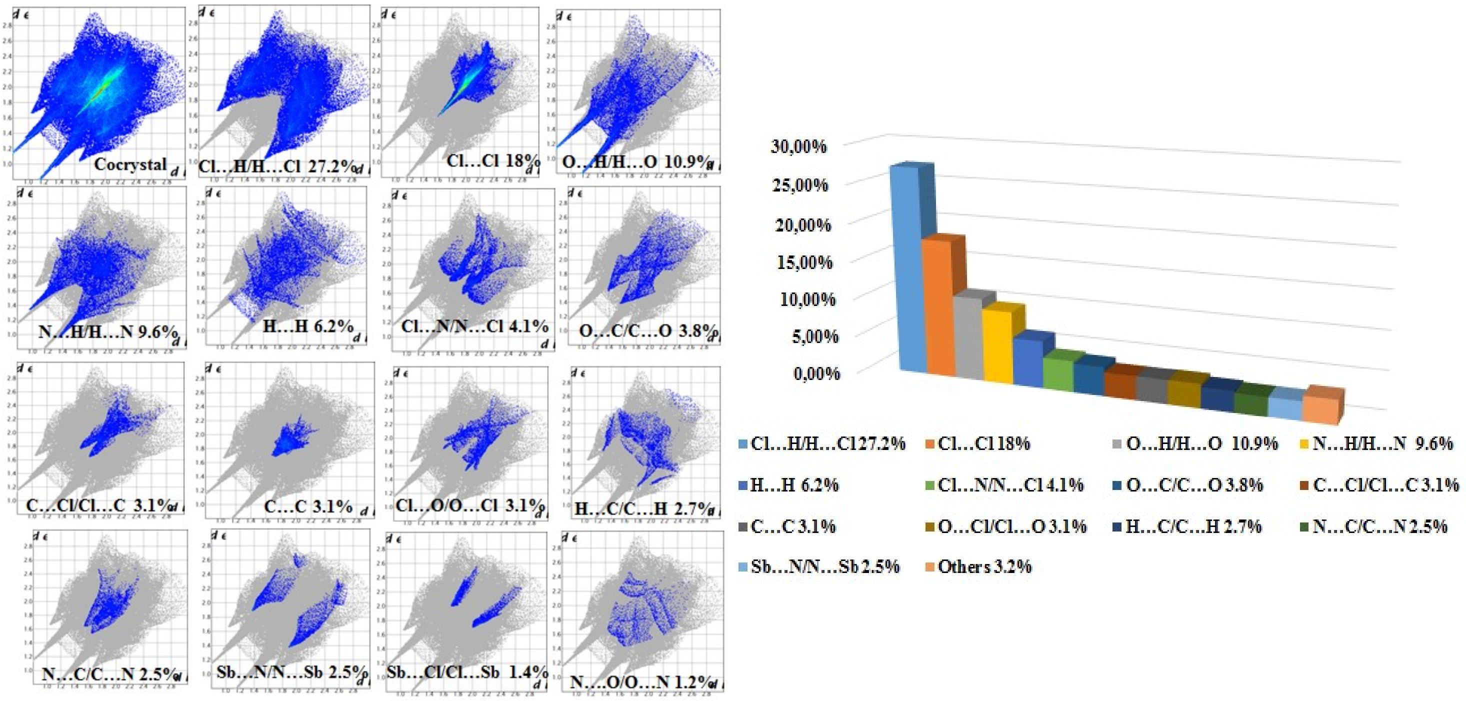

Fig. S7. 2D fingerprint plots of the cocrystal and relative contributions of differents intermolecular contacts to the Hirshfeld surface.

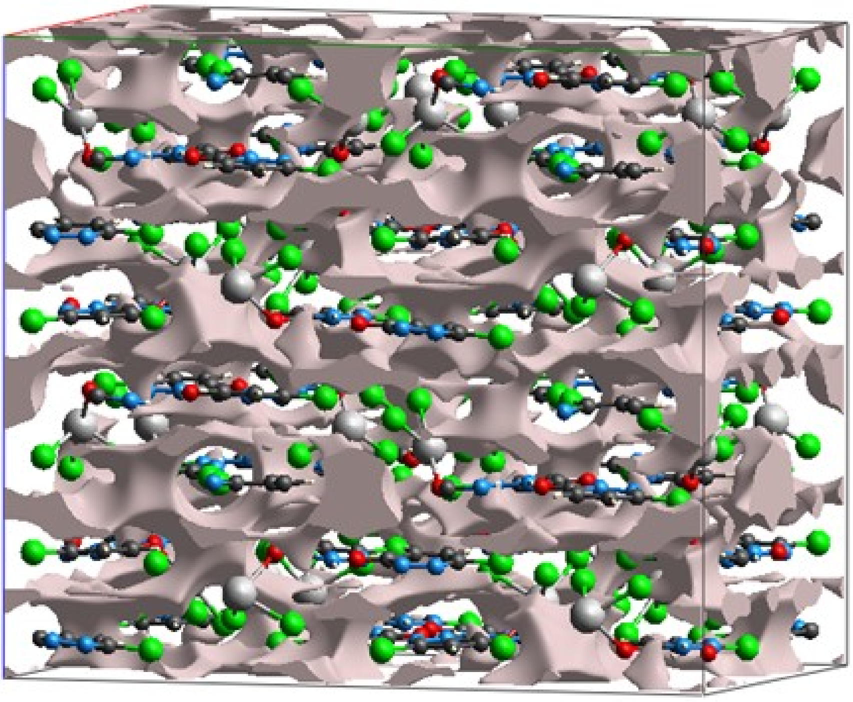

Fig. S8. 3D graphical view of voids in the crystal packing of the **PdzSb**.

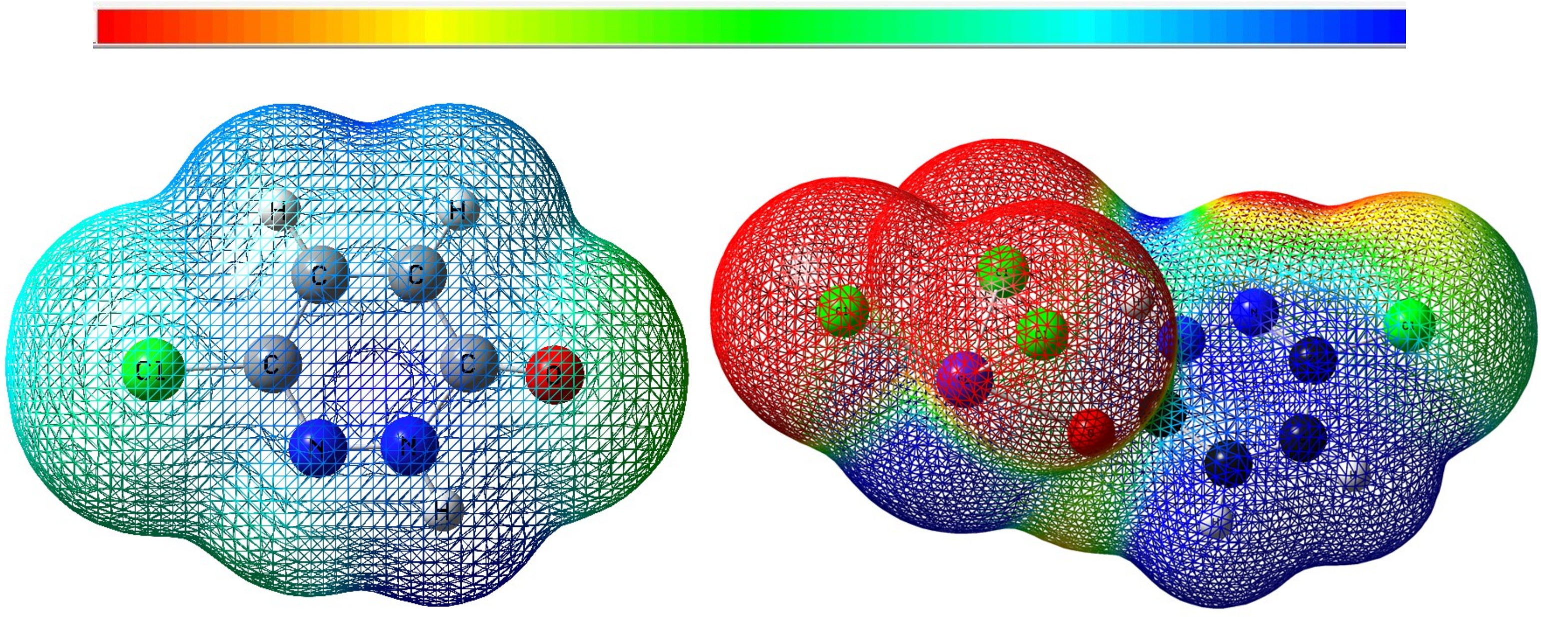

(a) **(b)**

Fig. S9. Molecular electrostatic potential (MEP) surfaces of the individual molecules (a) and (b), showing the localized charge distribution and the regions involved in intermolecular interactions. with values given in arbitrary units (a.u.).

Table S1: Selected bond lengths (Å) and angles (°) in anionic part of **PdzSb.**

Distances (Å)			
**Sb1—O1**	2.379 (5)	**Sb2— Cl5**	2.486 (18)
**Sb1—Cl2**	2.3822 (19)	**Sb2— Cl6**	2.3646 (18)
**Sb1—Cl3**	2.357 (2)	**Sb2— Cl7**	2.3727 (19)
**Sb1—Cl4**	2.493 (19)	**Sb2— O8**	2.385 (5)

Angles (°)			
**Cl3— Sb1—O1**	83.90 (15)	**Cl6— Sb2—Cl7**	91.99 (7)
**Cl3—Sb1—Cl2**	92.65 (8)	**Cl6— Sb2—O8**	84.16 (14)
**O1— Sb1—Cl2**	85.22 (14)	**Cl7— Sb2—O8**	84.77 (14)
**Cl3—Sb1—Cl4**	91.91 (7)	**Cl5— Sb2—Cl6**	92.10 (7)
**O1— Sb1—Cl4**	174.30 (8)	**Cl7— Sb2—Cl5**	91.62 (6)
**Cl2— Sb1—Cl4**	91.13 (7)	**O8— Sb2—Cl5**	174.69 (13)

Table S2: The attributions of calculated and observed frequencies of the vibration modes of the **PdzSb** Co-Crystal.

IR (cm^{-1})	Raman (cm^{-1})	Calc. Wavenumbers(cm^{-1})	Assignement
3456	-	3402	$\upsilon(N-H)$
3381	-	3391	$\upsilon_{as}(C-H) + \upsilon_s(C-H)$
3182	-	3198	$\upsilon(N-H)$
1680	1662	1673	$\upsilon(C-O) + \beta(N-H)$
1620	1605	1615	$\upsilon(C=O)$
1548	-	1537	$\upsilon(C=C)$
1437	1422	1433	$\delta(N-H)$
1298	1307	1283	$\upsilon(C-C)$
1228	1233	1237	$\upsilon(C-Cl) + \upsilon(C-N)$
1149	1155	1158	$\upsilon(N-N)$
1118	1122	1123	$\tau(C-H)$
998	-	990	$\omega(C-H)$
940	-	944	$\gamma(N-H)$
847	840	845	$\gamma(C-H)$
670	673	666	$\delta(N-C-C) + \delta(C-C)$
541	-	536	$\upsilon(Sb-O)$
-	416	400	$\upsilon_s(Sb-O)$
-	309	311	$\upsilon_s(Sb-O)$
-	255	260	$\upsilon_s(Sb-Cl)$
-	190	203	$\upsilon_{as}(Sb-Cl)$
-	166	168	$\delta(Sb-Cl)$
-	130	134	$\delta(Sb-Cl)$
	104	106	**Lattice Mode**

υ_s: symmetric stretching, **υ_{as}**: asymmetric stretching, **β**: in plane bending, **γ**: out plane bending, **δ**: scissoring, **ω**: wagging, **τ**: twisting

Table S3: Summary of Noncovalent Interactions and Corresponding Distances (Å) of the **PdzSb** Co-Crystal.

**Type of interactions**	**Distance (Å)**
π-π	3.732- 3.873
π-Cl	3.658- 3.834
Sb…Cl	3.430- 3.517
O…Cl	3.470- 3.475
Cl…Cl	3.213- 3.769
Sb…N	3.232
π-O	2986- 3588

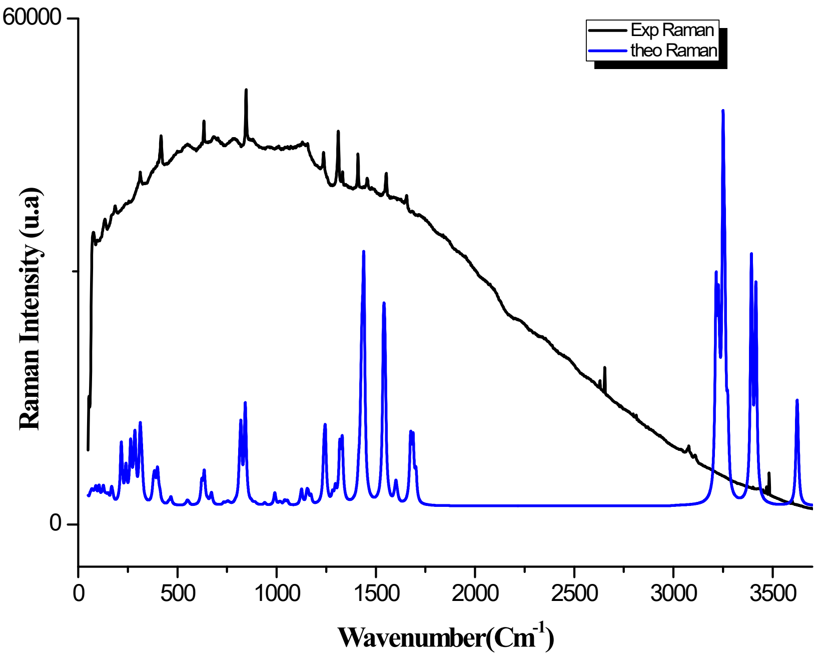

Fig. S10. Original Raman spectrum of the compound before background correction, showing the strong photoluminescence contribution.
